# Editorial: Fostering sustainable career throughout lifespan of employees

**DOI:** 10.3389/fpsyg.2023.1161624

**Published:** 2023-03-28

**Authors:** Asta Savanevičienė, Pascale Peters, Amelia Manuti, Carlos Cabral-Cardoso, Agnieszka Karman

**Affiliations:** ^1^School of Economics and Business, Kaunas University of Technology, Kaunas, Lithuania; ^2^Center for Strategy, Organization, & Leadership, Nyenrode Business Universiteit, Breukelen, Netherlands; ^3^Department of Organization, Leadership and Management, Inland Norway University of Applied Sciences, Lillehammer, Norway; ^4^Department of Education, Psychology, Communication, University of Bari “Aldo Moro”, Bari, Italy; ^5^Faculty of Economics, University of Porto, Porto, Portugal; ^6^Faculty of Economics, Marie Curie-Sklodowska University, Lublin, Poland

**Keywords:** sustainable career, career shock, positive experiences at work, HRM practices, physical and mental health

## 1. Introduction

Throughout the career lifespan, people increasingly experience shocks for various reasons, which might result in unsustainable careers. These may include work-life balance issues, increased workload, job losses, the need to adopt new technologies and new ways of working, for example due to the COVID-19 pandemic, and the need to strengthen one's physical and mental health. Therefore, a new paradigm and discourse on careers has gained popularity: “sustainable careers.” Inspired by “positive psychology,” this concept embraces an inclusive approach; it assumes that all employees have valuable competencies or talents that can be productively applied to organizations. Moreover, it highlights the relevance of positive experiences at work, attributed to human-centered HRM practices that allow employees to utilize their potential both at and outside of work, meanwhile ensuring physical and mental health.

This Research Topic unveils the topic of sustainable careers throughout the employee lifespan by exploring the interactions between stakeholders in developing sustainable careers, disclosing challenges for different sets of employees, while ensuring a sustainable career, explaining the interplay between the individual and the context for a sustainable career, and revealing the role of organizational and supervisory support in the sustainable career of employees.

The four overarching sub-themes presented in the six papers in this Research Topic can be summarized as follows.

## 2. Toward conceptual development of a sustainable career

Hallpike et al. presented a discourse on individuals being key-drivers in shaping their own sustainable careers and the context providing supports or barriers to the pursued career trajectory. The authors suggested a distributed interactive career decision-making framework (diCDM) in which both the individual and the context are responsible for the development of sustainable careers. In their view, career decisions depend on the interaction between individual and their career contexts over time, in a system of distributed agency. This is especially relevant in the context of ever-changing labor markets, globalization, technological development, retirement policies, and the global COVID-19 pandemic, which has erased the boundaries between work and personal life.

## 3. Challenges for different sets of employees while ensuring a sustainable career

Individuals' characteristics may lead to different expectations and challenges and distinctive understandings of “sustainable career,” which organizations need to consider to retain them. Tang et al., for example, drew attention to the outset of sustainable careers. Based on empirical data from 247 newcomers, they highlighted the issue of work stress during the organizational socialization process that affected the socialization process both negatively and positively.

Karlsen et al., in contrast, revealed how organizations ensured the sustainable careers of later-life employees, by focusing on Selection, Optimization, and Compensation (SOC) strategies across organizational levels to ensure a healthy and high-quality working life. Based on 23 semi-structured interviews with senior employees, the authors identified specific manifestations of SOC strategies across all organizational levels. Strikingly, at the leadership level, compensation strategies were not found.

## 4. The interplay between the individual and the context for a sustainable career

Personal resources can strengthen the role of individuals in sustainable career decision-making, while context can create support or barriers. While discussing the topic of skills and abilities to thrive in remote work, Henke et al. uncovered a link between individuals and their contexts when striving for sustainable careers. More specifically, the authors studied how individuals reacted to the sudden shift to remote work due to the pandemic and, consequently, how organizations had to create remote working arrangements and physical conditions. Based on 59 semi-structured interviews, they revealed that the ability to manage virtually and technology literacy were critical while ensuring remote work. Meanwhile, the organization's obligation was to provide the necessary technology and equipment and to improve the IT support. Special attention should have been paid to shifting the culture to be more supportive of remote working and ensuring transparent communication. Only goal-oriented decisions of all stakeholders made it possible to ensure sustainable careers.

## 5. The role of organizational and supervisory support in sustainable careers

Almost all articles emphasized the role of organizational and supervisory support in a sustainable career of employees. In their literature review on the relationship between job involvement, perceived organizational support (POS), and organizational commitment with job insecurity, Hngoi et al. revealed that most research articles focus on the relationship between job insecurity and organizational commitment. Several articles, however, also examined the relationship between job involvement and job insecurity and the linkage between POS and job insecurity. Nevertheless, the authors highlighted the lack of studies on the antecedents of job insecurity. Henke et al. emphasized the relevance of managerial support and that employees want to feel that their wants and needs are considered, particularly in hybrid work. Tang et al. disclosed that managerial support can turn a competitive psychological climate into a motivating stressor, eliminating it as a barrier for subjective and objective career success. Using survey data of 260 employees from nine companies, Wang et al., showed that abusive supervision can enhance work–family conflict, but that this can be reduced by strong optimism due to higher endurance in high-pressure situations, which enables employees to achieve their planned career goals. They revealed the individual-context interplay, demonstrate the compensation mechanisms, and explain their mutual interactions.

## 6. Conclusion and future research

We hope that this Research Topic on sustainable careers will encourage future research to deepen our understanding of the phenomenon and provide new insights for practitioners. We suggest future research to:

- Extend theoretical conceptualizations, mechanisms, and models on sustainable careers and their operationalizations;
- Conduct empirical research to provide deeper insights by considering both the diversity of individual-related characteristics and the abundance of context-specific factors;
- Enhance understanding of the motives behind people's career decisions and how these change over time, by examining these across multiple life stages, genders, socio-economic status groups, et cetera;
- Examine the interplay between the context and different sets of employees, especially paying attention to more vulnerable groups;
- Continuously review individual-context interactions for ensuring sustainable careers in a rapidly changing environment, characterized by technological breakthroughs and associated new work models.

## Author contributions

AS, PP, AM, CC-C, and AK are co-editors for this Research Topic. All authors contributed to the article and approved the submitted version.

